# The tryptophan kynurenine pathway, neopterin and IL-6 during vulvectomy and abdominal hysterectomy

**DOI:** 10.1186/s12929-014-0102-2

**Published:** 2014-12-20

**Authors:** Jaap Willem Hol, Robert J Stolker, Markus Klimek, Dirk L Stronks, Durk Fekkes

**Affiliations:** Department of Anesthesiology, Erasmus Medical Center, PO Box 2040, 3000 CA Rotterdam, The Netherlands; Department of Clinical Chemistry, Erasmus Medical Center, Rotterdam, the Netherlands

**Keywords:** Kynurenine, Tryptophan, Indoleamine 2,3-dioxygenase, Neopterin

## Abstract

**Background:**

Surgery has wide ranging immunomodulatory properties of which the mechanism is poorly understood. In order to investigate how different types of surgery influence inflammation, we designed a longitudinal observational study investigating two inflammatory profiles of two separate patient groups undergoing gynaecological operations of differing severity. In addition to measuring the well known inflammatory markers neopterin and IL-6, we also determined the kynurenine/tryptophan ratio.

This study was a prospective, single center, two-armed observational study involving 28 female patients. Plasma levels of tryptophan, kynurenine, neopterin and IL-6 were determined from samples taken at: 24hrs pre-operative, prior to induction, ten minutes before the operation was expected to end, and at 24 and 96 hours post operative in patients undergoing abdominal hysterectomy and vulvectomy.

**Results:**

There were 15 and 13 patients included in the vulvectomy and abdominal hysterectomy groups, respectively. In this study we show that anesthesia and surgery significantly increases the enzyme activity of indoleamine 2, 3 dioxygenase (IDO) as measured by the kynurenine/tryptophan ratio (P=0.003), while maintaining stable neopterin levels. However, abdominal hysterectomy causes a considerable IL-6 increase (P<0.001).

**Conclusion:**

Surgery and associated anesthesia cause a significant tryptophan level decrease while significantly increasing IDO activity. Both types of surgery produce nearly identical neopterin time curve relationships, with no significant change occurring in either group. However, even though neopterin is unaffected by the severity of surgery, IL-6 responded to surgical invasiveness by revealing a significant increase during abdominal hysterectomy.

## Background

Surgery has wide ranging immunomodulatory properties of which the mechanism is poorly understood [1-3]. In order to better understand the effect of surgery and anesthesia on inflammation, we designed a longitudinal observational study investigating two inflammatory profiles of two separate patient groups undergoing surgery of differing severity while undergoing general anesthesia. In addition to measuring the well known inflammatory markers neopterin and IL-6, we also determined kynurenine and tryptophan.

Tryptophan is an essential precursor for serotonin and kynurenine (KYN) [4]. The metabolism of tryptophan to kynurenine is facilitated by the enzyme Indoleamine 2,3 dioxygenase (IDO) [5]. Tryptophan is a vital amino acid for growth. When present in limited amounts it inhibits viral, bacterial and parasitic development. Not only are microorganisms dependent on tryptophan, but T-cell production is also limited by decreased levels [6-8]. Kynurenine, the direct product of tryptophan catabolism has several important physiological and immunosuppressive properties. It regulates immune function by suppressing T-cells and natural killer cells [9,10]. Moreover, recent research has found that it contributes to arterial vessel wall relaxation and causes hypotension in a dose dependent manner in systemically inflamed mice [11].

**Figure 1:**
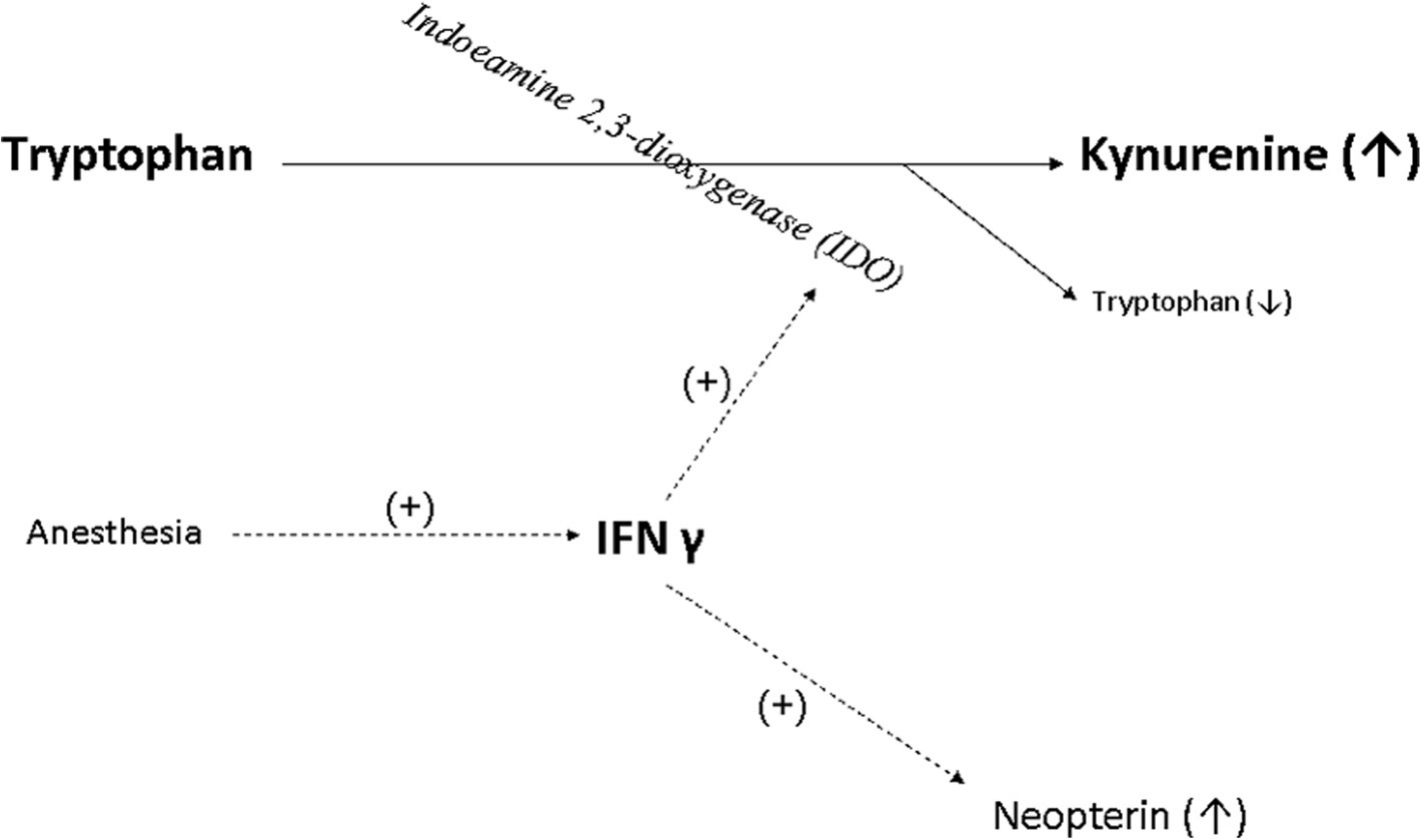
**An overview of the tryptophan kynurenine pathway and its relationship with IFN γ as well as neopterin.** (+) denotes that it activates and or stimulates production.

The tryptophan kynurenine pathway is regulated by the rate limiting enzyme IDO [5,12] (Figure 1). IDO is made in the vascular endothelial cells and is activated via autocrine and paracrine mechanisms by means of interferon γ (IFN γ) released by dendritic and T-cells [13,14]. The amount of kynurenine produced relates to its activity [12,15]. In addition, IDO contributes to the regulation of blood pressure because it controls the production of kynurenine. This has been demonstrated by the ability of IDO inhibitors to restore normal systolic blood pressure in septic mice [11].

Tryptophan degradation is correlated to neopterin formation; both are stimulated by IFN γ [4,16-19]. Increased neopterin levels are associated with a pro-inflammatory state mediated by cellular immune system activation [20-22]. It is an endogenous regulator of cytotoxic effects by activated macrophages and a potent enhancer of peroxynitrite [23]. It is a stable molecule eliminated only by the kidney. Higher levels are associated with higher levels of reactive oxygen species and thus serve as an estimate of the oxidative stress caused by the immune system [24,25]. Levels of neopterin have been shown to predict the development of septic complications in trauma and post surgical patients. There is a direct correlation between increased neopterin levels and non-survivors [26].

IL-6 is an acute phase pro-inflammatory cytokine capable of being released by macrophages, endothelial cells and T-cells minutes after injury. It is a good marker for acute immune system activation and is accepted as a sensitive early marker of tissue damage with peak serum levels being proportional to the amount of surgical trauma [27-30]. Known to be made within minutes after injury, it is also broken down in a relatively short amount of time [31,32].

Quantifying the above mentioned biochemical substances in blood may give us more insight into peri-operative immunomodulatory processes. Significantly different inflammatory profiles can have wide clinical implications. We hypothesize that more invasive surgery when compared to minor surgery causes significantly more increases in all inflammatory biomarkers measured.

## Methods

### Study set-up and inclusion criteria

This study was a prospective, single centre, two-armed observational study with 28 female patients. The protocol was approved by the Medical Ethics Committee of the Erasmus Medical Centre, Rotterdam (MEC-2008-134). All procedures were performed in accordance with the Helsinki declaration. Informed consent was obtained from all patients.

Inclusion criteria were 1) scheduled for vulvectomy or abdominal hysterectomy, 2) expected surgery duration greater than 0.5 h, 3) postoperative hospitalization lasting more than 4 days, 4) age greater than 18 years, 5) ASA (American Society of Anesthesiologists) classification I-III. Exclusion criteria were 1) ASA-classification IV-V, 2) patients unable to speak Dutch, 3) and patients not able to consent. Patients had the right to withdraw from the study at any time. Patients who developed serious adverse side effects were to be withdrawn from the study.

### Anesthesia procedure

All patients received 1.0 mg tablet lorazepam and 100 mg celecoxib (selective COX-2 inhibitor) approximately one hour before surgery. Personal drug regimens were continued during the study. The observational nature of this study allowed the staff anesthesiologist to place an epidural catheter if the anesthesiologist felt it was indicated for adequate post operative pain control. All patients received total intravenous anesthesia, using propofol for sedation and sufentanil for analgesia. Cisatracurium provided muscle relaxation for patients being intubated. Prior to the first incision all patients received antibiotics (1 g cefazoline and 500 mg metronidazol).

For all patients the minimum post operative pain control regimen included 4000 mg paracetamol and 200 mg celecoxib per 24 hours. While in the recovery room, morphine was titrated until sufficient pain control was achieved. The daily regimen of paracetamol and celecoxib was continued until patients no longer experienced pain with a VAS greater than four. Patients with an epidural catheter had it removed when the anesthesiologist determined that it was no longer indicated for adequate pain control.

### Outcome measures

Patient demographics, medications used during and after surgery, and duration of surgery were documented. EDTA blood samples (4 ml) were collected for determination of neopterin, kynurenine, and tryptophan at 24 hours pre-operative, right after IV placement prior to induction, ten minutes before anesthesia was expected to end, and at 24 and 96 hours post operative. Plasma was isolated by centrifugation at 2650 gmax for 20 minutes at 20°C; samples were stored at -80°C until assay. At the same time points blood was collected for preparation of serum used for the measurement of IL-6.

Concentrations of kynurenine and tryptophan were determined via their natural fluorescence using an isocratic, reversed-phase HPLC system (Agilent) and an FP-2020 fluorescence detector (Jasco) as described previously [33]. The analytical column consisted of a 250 × 2.1 mm i.d. column packed with 5 μm particles of GraceSmart RP-18 (Grace Davison Discovery Sciences), which was protected by a guard cartridge column (4.0 × 2.0 mm i.d.) containing Phenomenex C18 material. An HP ChemStation (Hewlett Packard) was used for data collection and handling. The kynurenine/tryptophan ratio was calculated to estimate the activity of the enzyme indoleamine 2,3 dioxygenase (IDO) [34].

Total neopterin was measured after acid oxidation. Plasma (0.4 ml) was oxidized in 0.1 ml 1 M trichloroacetic acid and 0.05 ml iodine solution (0.5% I_2_, 1% KI in 0.2 M trichloroacetic acid). After standing for 60 min under reduced light, excess iodine was reduced by the addition of 20 μl of 1% ascorbic acid solution and the mixture was centrifuged at 12 000 × g for 15 min at 4°C. The supernatant (0.4 ml) was transferred to an amber glass vial and 10 μl was injected directly onto the analytical column using an HPLC system with an auto sampler and a fluorescence detector as described previously [35].

Enzyme immunoassays for the quantitative determination of human IL-6 were performed with a sandwich ELISA (Pelikine Compact™ and additional Pelikine Toolset™, Sanquin, Amsterdam, The Netherlands) as described previously [36]. Data were calculated as pg/ml. IL-6 Figures were made with Sigma Plot, version 9.0. The results are presented as mean ± the standard error of the mean (SEM).

### Statistical analysis

Data was analyzed using SPSS for windows, version 16.01. The independent sample t-test was used to compare means for patient demographics (excluding ASA classification) and peri-operative characteristics. The Pearson Chi-square test was used to evaluate differences in ASA classification. The Fisher exact test was used to analyze difference in the type of pain control techniques used between groups (NSAID only, NSAID + Opiates, NSAID + Opiates + Epidural). All data are reported as the mean ± SD.

Our biochemical data were analyzed using MANOVA. Differences in values measured between the experimental groups across all time points and interaction between experimental groups and time were analyzed using multivariate repeated measures. Experimental group and time were the independent variables. When Mauchly’s Test of Sphericity was significant, the Greenhouse-Geisser test was used.

When a significant difference was found between experimental groups a one-way ANOVA test with post-hoc multiple comparisons (Bonferroni correction) was used to analyze the relationship between the level of the amino acid from the first pre-operative measurement until 96 hours post operative. The same Bonferroni correction was employed to analyze differences between experimental groups and time. Pairwise comparisons were used to analyze significant differences between longitudinal time points. P-value <0.05 was considered statistically significant.

## Results

### Demographics

Twenty eight female patients were included in the study; no patients were withdrawn from the study (Table 1). The vulvectomy group contained 15 patients, while the laparotomy group contained 13 patients. Significant intergroup differences were found for age and ASA classification (P < 0.05). Laparotomy patients were younger and had significantly lower ASA scores.

**Table 1:** **Patient demographics**

	**Vulvectomy**	**Laparotomy**
Age (years)	62 ± 12	44 ± 9^*^
Height (cm)	166 ± 5	167 ± 6
Weight (kg)	71 ± 10	71 ± 7

Data are mean ± SD.

*Significant difference between groups, P < 0.001.

### Perioperative characteristics

Differences in perioperative characteristics were found (Table 2). The abdominal hysterectomy group had a significantly longer operating time (P = 0.002), more blood loss (P < 0.001) and correspondingly more fluid replacement in the form of colloids (P = 0.002) and crystalloids (P = 0.005). In addition, significantly more propofol was used (P = 0.026), however, there was no significant difference in the amount of sufentanil used between groups. One of the patients in the laparotomy group received a blood transfusion with 285 ml of erythrocytes.

**Table 2:** **Perioperative characteristics**

	**Vulvectomy**	**Laparotomy**
Propofol during operation (mg)	1146 ± 828	1983 ± 105*
Operation time (min)	126 ± 50	188 ± 46*
Blood loss during operation (ml)	134 ± 271	959 ± 335ª
Colloids during operation (ml)	170 ± 242	540 ± 335*
Crystalloids during operation (ml)	1182 ± 627	1917 ± 655*
Sufentanil during operation (μg)	33 ± 15	37 ± 14
Cisatracurium during operation (mg)	20 ± 25	68 ± 18*

Data are mean ± SD.

*Significant difference between groups, P < 0.05.

ªSignificant difference between groups, P < 0.001.

There was no significant difference in the amount of post operative morphine, paracetamol or celecoxib given to both groups (Table 3). However, 9 patients from the laparotomy group were given pre-operative epidural catheters while only 3 patients were given one in the vulvectomy group. All epidural catheters were removed by 24 hours post–operative because pain control was found to be adequate.

**Table 3:** **Postoperative characteristics**

	**Vulvectomy**		**Laparotomy**	
	**24 hours post-operative**	**96 hours post-operative**	**24 hours post-operative**	**96 hours post-operative**
Paracetamol (mg)	3769 ± 832	4000 ± 0	3833 ± 577	4000 ± 0
Celecoxib (mg)	200 ± 0	186 ± 38	200 ± 0	233 ± 82
Morphine (mg)	6 ± 2	-	12 ± 1	-

Data are mean ± SD. Celecoxib (selective COX-2 inhibitor).

### Plasma tryptophan levels

**Figure 2:**
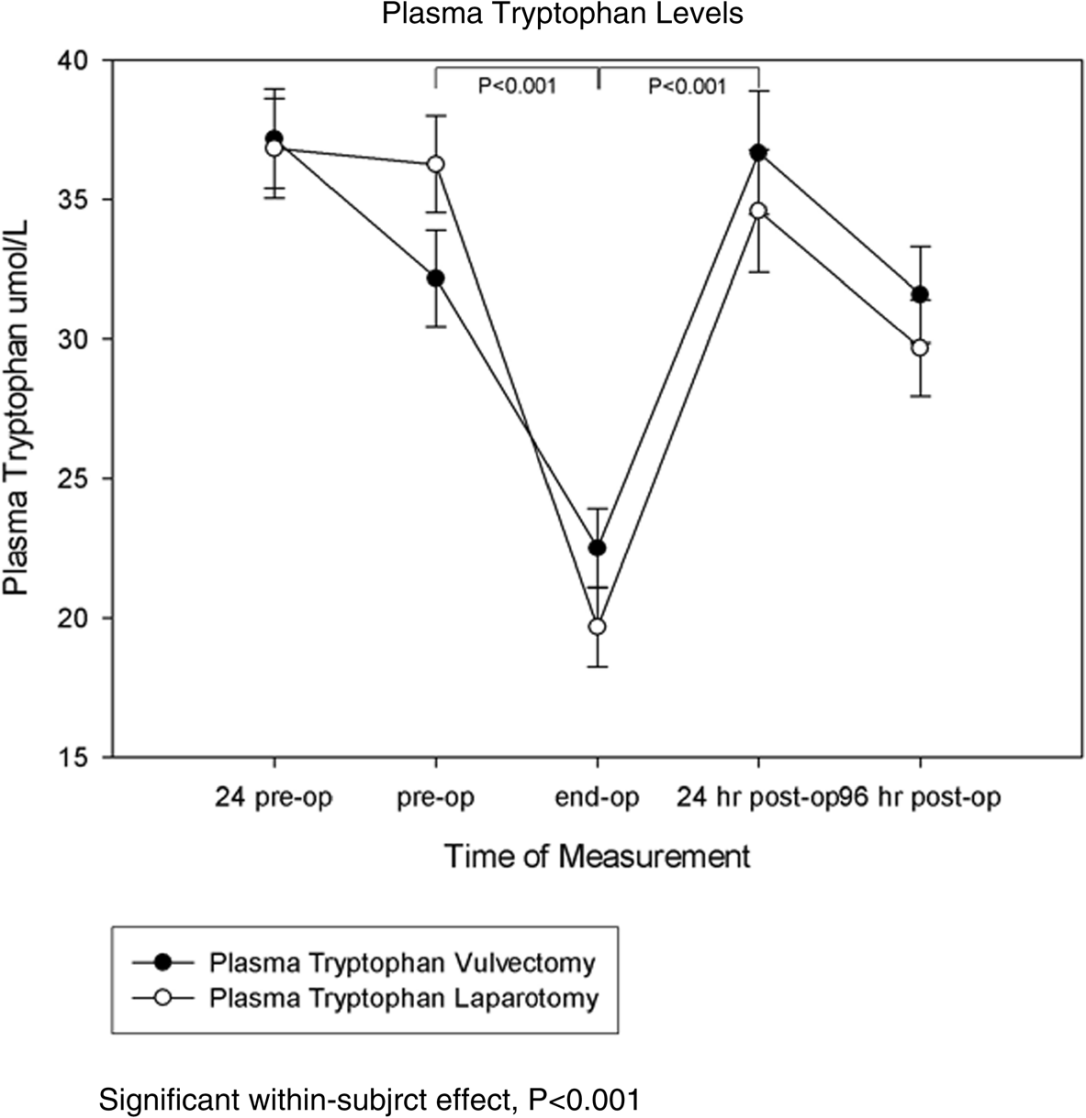
**The effect of anesthesia in patients undergoing vulvectomy and major abdominal surgery on perioperative levels of plasma tryptophan.** Values are the mean and SEM. There was a significant within subject effect (P < 0.001). There was a significant decrease prior to induction and 30 minutes prior to the end of operation ( bracket P < 0.001). A significant increase occurred 30 minutes prior to the end of operation and 24 hours post operative (bracket P < 0.001).

Baseline levels of plasma tryptophan 24 hours before surgery did not significantly differ between groups. Throughout time, no significant difference between experimental groups was found. There was however a significant within subject effect over time (P < 0.001) (Figure 2).

Pairwise comparisons reveal a significant decrease between plasma tryptophan measured prior to induction and thirty minutes prior to the end of the operation (P < 0.001). Following this significant decrease there was a significant increase between plasma tryptophan measured thirty minutes prior to the end of the operation and plasma measured 24 hours post operative (P < 0.001).

### Plasma IDO activity, defined by the ratio of tryptophan/kynurenine

**Figure 3:**
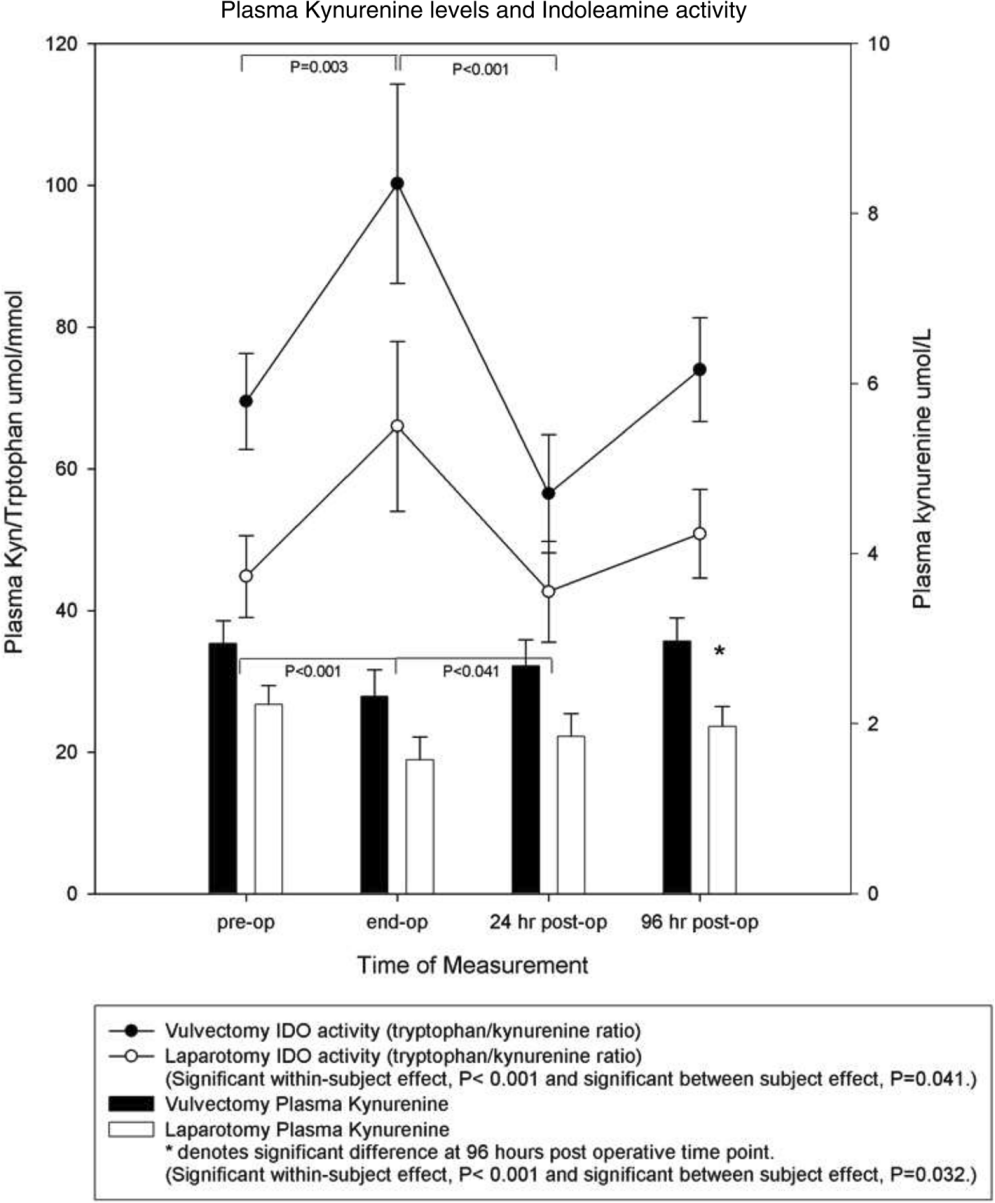
**Line graph.** The effect of anesthesia in patients undergoing vulvectomy and major abdominal surgery on perioperative IDO activity as defined by the ratio of tryptophan/kynurenine. Values are the mean and SEM. There was a significant within subject effect (P < 0.001). In addition, a significant difference between experimental groups was found (P = 0.041). There was a significant increase in plasma IDO activity measured prior to induction and thirty minutes prior to the end of the operation (bracket P = 0.003). A significant decrease occurred between IDO activity measured prior to the end of the operation and levels measured at 24 hours post operative (bracket P < 0.001). *Bar graph:* The effect of anesthesia in patients undergoing vulvectomy and major abdominal surgery on perioperative levels of KYN. Values are the mean and SEM. There was a significant within subject effect (P < 0.001) as well as a significant difference between experimental groups (P = 0.032). At 96 hours post operative, the laparotomy group had significantly lower levels of plasma KYN than the vulvectomy group (P = 0.028). There was a significant plasma KYN level decrease during measurements made prior to induction and the end of operation (bracket P < 0.001). A significant increase occurred between points measured prior to the end of operation and 24 hours post operative (bracket P < 0.041).

Baseline levels of plasma IDO activity were not significantly different between both groups. There was a significant within subject effect over time (P < 0.001). In addition, a significant difference between experimental groups was found (P = 0.041) (Figure 3).

Pairwise comparisons reveal a significant increase in plasma IDO activity measured prior to induction and thirty minutes prior to the end of the operation (P = 0.003). Following this significant increase there was a significant decrease between plasma IDO activity measured thirty minutes prior to the end of the operation and plasma measured 24 hours post operative (P < 0.001).

### Plasma kynurenine

Baseline levels of plasma kynurenine were not significantly different between both groups. There was a significant change in plasma kynurenine levels over time for both groups (P < 0.001) as well as a significant difference between experimental groups (P = 0.032). At 96 hours post operative, the vulvectomy group had significantly higher levels of plasma kynurenine than the laparotomy group (P = 0.028) (Figure 3).

Pairwise comparisons reveal a significant decrease in plasma kynurenine measured prior to induction and thirty minutes prior to the end of the operation (P < 0.001). Following this significant decrease there was a significant increase between thirty minutes prior to the end of the operation and plasma measured 24 hours post operative (P < 0.041).

### Plasma neopterin levels

**Figure 4:**
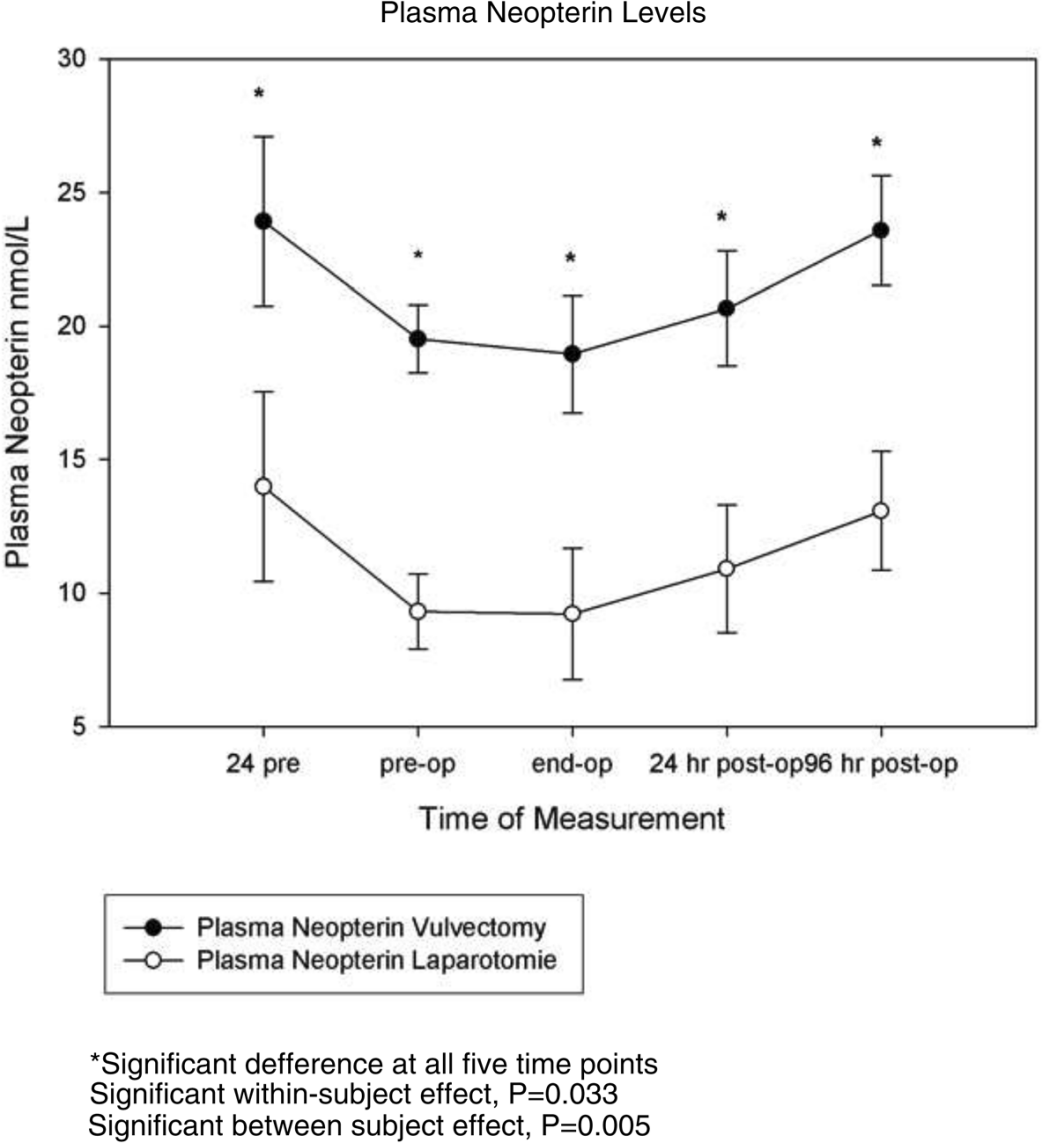
**The effect of anesthesia in patients undergoing vulvectomy and major abdominal surgery on perioperative levels of plasma Neopterin.** Values are the mean and SEM. There was a significant within subject effect (P < 0.033) and a significant difference between groups (P = 0.005).

Baseline levels of plasma neopterin significantly differed between both groups (P = 0.002); the vulvectomy group having much higher levels than the laparotomy group. A significant plasma neopterin level change occurred over time for both groups (P < 0.033). There was also a significant difference between experimental groups (P = 0.005) (Figure 4).

### Plasma IL-6 levels

**Figure 5:**
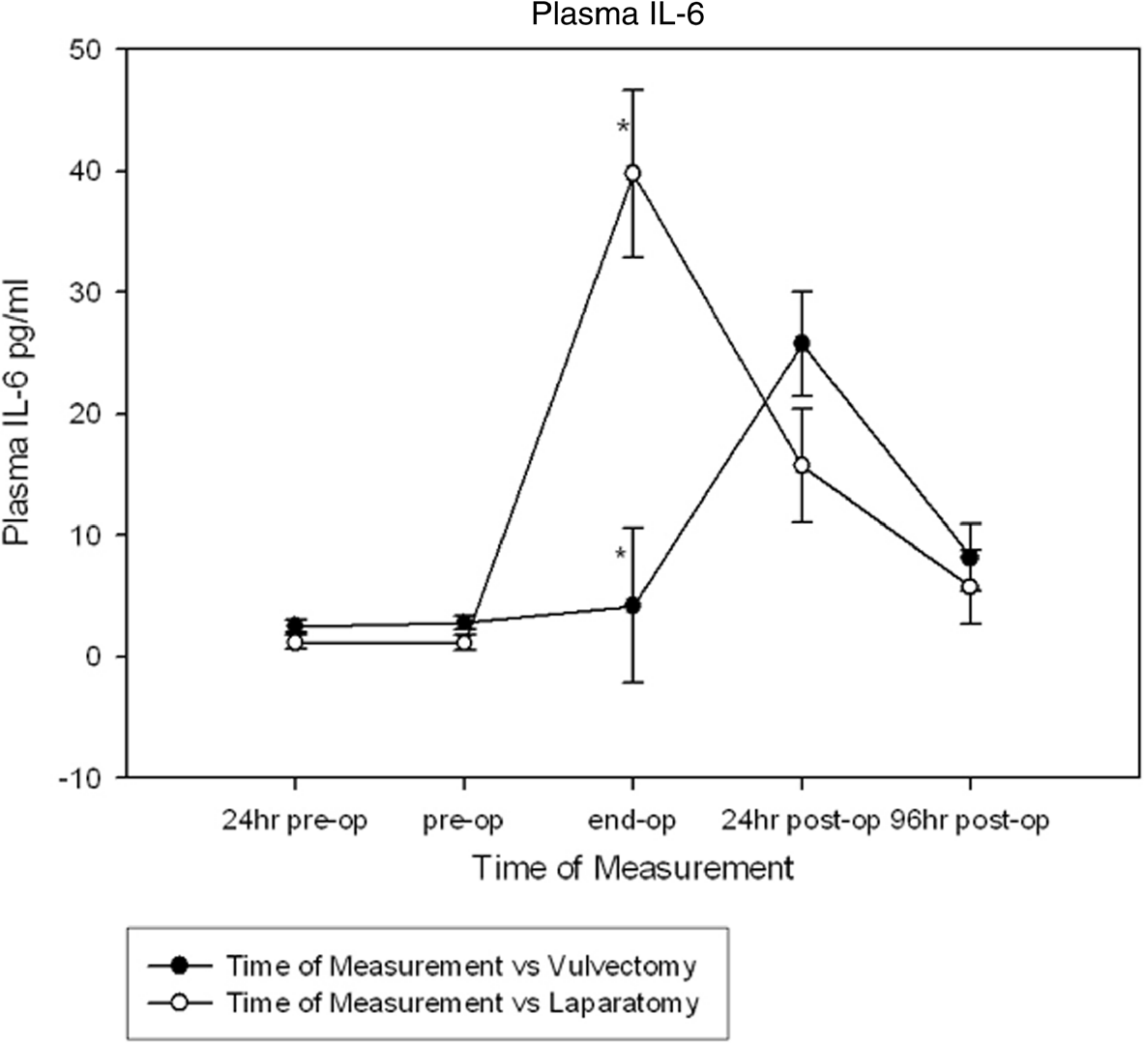
**The effect of anesthesia in patients undergoing vulvectomy and major abdominal surgery on perioperative levels of plasma IL-6.** There was a significant within subject effect (P < 0.001). The major abdominal surgery group had produced significantly more IL-6 then the vulvectomy group 30 minutes prior to the end of the operation (P < 0.001). *Denotes significant difference at the end of operation time point, P<0.001.

Baseline levels of plasma IL-6 were not significantly different. A significant plasma IL-6 level change over time occurred for both groups (P < 0.001). There was no significant difference between groups. However, the major abdominal surgery group had produced significantly more IL-6 than the vulvectomy group 30 minutes prior to the end of the operation (P < 0.001) (Figure 5).

## Discussion

In this study we show that surgery and associated anesthesia significantly increase IDO activity, while maintaining stable neopterin levels. However, abdominal hysterectomy causes a major IL-6 increase. Via this longitudinal observational study, we believe we are the first to compare how two well defined surgical interventions of differing severity affect the tryptophan kynurenine pathway and the two inflammatory mediators neopterin and IL-6. It must be stressed, that there are differences in age and ASA-score between the groups. It was not our intention to compare two more or less identical groups. Due to the fact that the higher age and the higher ASA-score are associated with the vulvectomy group while most changes occur in the younger and healthier abdominal hysterectomy group, we can conclude that the observed effects are caused by the procedure and not by comorbidity.

Our tryptophan results show that in both groups stable baseline levels significantly decrease after induction with a significant rebound occurring during the 24 hour period after surgery. This data suggest that tryptophan is being rapidly consumed after induction. Anesthesia has been shown to cause IFN gamma release which in turn activates the enzyme IDO [37-39]. Our results show that there is significant activation (P = 0.003) of the IDO enzyme as suggested by the tryptophan/kynurenine ratio measured in both groups. During the 24 hours following surgery, however, a significant deactivation of IDO occurs (P < 0.001), although 96 hours after surgery IDO activity returns to pre-operative levels. The significant kynurenine increase seen between the end of operation and 24 hours post operative might provide an explanation for the corresponding drop in IDO activity. Allowing for a negative feedback mechanism capable of maintaining KYN homeostasis, KYN suppresses the activation of T cells and NK cells which are responsible for IFN gamma secretion, one of the known factors responsible for activating IDO.

Neopterin is primarily produced by human monocytes and macrophages. Increased neopterin concentrations are indicative of cellular immune activation [19]. We found significantly higher levels of neopterin at all five time points in the vulvectomy group. The cancer necessitating vulvectomy may explain these higher levels because cancer has been correlated with higher neopterin levels [25,40]. It is clearly evident that the time curve relationship is nearly identical for both groups. Remarkably, anesthesia and surgery did not significantly dampen or increase neopterin levels. Instead, there seems to be a neopterin level drop during the 24 hour period prior to induction with a slower recovery to baseline level occurring during the 96 hour time period after surgery.

Not surprisingly, patients awaiting surgery suffer high levels of anxiety [41]. There is good evidence that in healthy individuals, acute psychological stress causes increased levels of catecholamines and cortisol, which in turn modulate the immune system [42-45]. We speculate that increased stress and associated increased cortisol synthesis prior to surgery caused a neopterin level decrease in the 24 hours prior to anesthesia. Opiod based anesthesia, however, is known to suppress cortisol synthesis and this may explain why neopterin levels do not continue to drop after induction of anesthesia [38,46]. Another explanation may be that the decrease in neopterin is counteracted by an increased release of neopterin induced by IFN γ.

Unlike our neopterin results, there are barely measurable IL-6 levels during the 24 hours prior to surgery. As would be expected from an acute phase pro-inflammatory cytokine known to be a sensitive early marker of tissue damage, there was a sharp increase resulting in significantly more IL-6 being made in the major abdominal surgery group at the end of the operation compared to the vulvectomy group. It is noteworthy that at 96 hours post operative IL-6 levels almost return to baseline levels in both groups. The high prevalence of mast cells in the abdominal cavity might explain why an earlier and overwhelmingly more significant amount of IL-6 is released during the abdominal operation. A recent study provides convincing evidence that intestinal handling during open gynecological surgery causes mast cell activation and associated release of IL-6 and other inflammatory mediators [47]. When comparing with neopterin results it is reasonable to conclude that the large IL-6 peak found in the major abdominal surgery group is due to the more severe surgical trauma and intestinal handling experienced by this group.

The course of the tryptophan kynurenine pathway measured in both groups suggests that activated IDO might buffer overzealous cellular immune system activation by allowing for the restoration of KYN levels [38]. The trend seen in our neopterin results supports this idea. While our IL-6 results confirm that major abdominal surgery causes a more acute activation of the cellular immune system when compared to the less invasive vulvectomy group, the flare up seen in the major abdominal surgery group is short lived. This is confirmed by all of the data in this study. Between the end of the operation and 24 hours after surgery, the IL-6 peak quickly recedes to the same level seen in the less invasive (vulvectomy) group, while a significant kynurenine increase is seen. During this time frame, neopterin is still depressed when compared to baseline and 96 hour post operative levels.

In addition to its ability to suppress natural killer and T cells, kynurenine also has a vasodilating effect that might explain our results. It is likely that the vasodilating properties of propofol help depress kynurenine levels after induction. A recent study using animal models has found that kynurenine plays a similarly important role in determining arterial wall tonus as nitric oxide does [11]. The two mediators act redundantly to influence vascular tone [48-50].

Although this study is primarily intended to study the effects of differing degrees of surgery on inflammatory markers, this study also provides data that can be interpreted to show that anesthesia does not seem to be strictly immunosuppressive, but might have a buffering effect. It seems that adequate anesthesia preserves proper immune function while at the same time suppressing pathological immune activation in operations like major abdominal surgery which are more prone to a systemic inflammatory response syndrome (SIRS). Further research is necessary in order to investigate how opiates, hypnotic agents and the epidural technique specifically contribute to these effects. Most evidence suggests that opiates have immuno-modulating properties, while propofol allows for proper function of the immune system [51-55]. It is noteworthy that in this study there was significantly more propofol used in the group expressing a significant IL-6 increase. Taking this into account, it is likely that propofol is not responsible for immunosuppression.

### Limitations

The significantly less amount of propofol given to the vulvectomy group can be partly explained by the shorter operating time. However, women in this group were older and had higher ASA scores also explaining why less propofol was used. In addition, the greater amount of colloids and crystalloids given to the laparotomy group is related to the longer operating time and increased amount of blood loss in this group of patients. Nevertheless, the results shown in our figures make it clear that dilutional phenoma are not responsible for trends seen. Likewise, these peri-operative characteristics provide additional evidence that abdominal hysterectomy is a heavier, more traumatic operation in comparison to the vulvectomy procedure. The more severe nature of abdominal hysterectomy also explains why anesthesiologists used significantly more invasive analgesia techniques during the 24 hour post operative period as seen in Table 4. Generally, a heavier regimen was chosen for heavier surgery. According to our results this seems to have an appropriate effect because IL-6 only showed one moment where levels were different between groups at 24 hours post operative.

**Table 4:** **Analgesia technique applied 24 hours post operative**

	**Only NSAIDs**	**NSAIDs and intravenous morphine**	**NSAIDs, intravenous morphine and epidural anesthesia**
Vulvectomy	8	4	3
Laparotomy*	1	3	9

*Laparotomy group had significantly more invasive pain control, P < 0.05.

Furthermore, we did not consider or note the menopausal status of women when conducting this study, however, if the menopausal status had a confounding effect it would make our results even stronger: From the literature we know, that after menopause, there is an increase in pro-inflammatory serummarkers (IL-6 and TNF-alpha) [56]. When taking age into account, our vulvectomy group would have had more women who were post-menopausal than in the abdominal hysterectomy group. However, we found the higher IL-6-levels in the abdominal hysterectomy, the group with the younger patients. This supports the claim that the effect of the procedure is stronger than changes due to aging.

One might also discuss, whether it is prudent, to use COX-2-inhibitors as analgetics in a study on inflammation processes. We decided to do so, because it reflects our clinical standard practice and it would be considered an ethical problem by our institutional Medica Ethics Committee to change our pain control procedures just to create better study conditions. There is growing evidence that the suppression of inflammatory processes by COX-2-antagonists has more than only analgetic effects: depression and the immune response in cancer patients are two of the most interesting fields [57-59]. We know that COX-2-inhibition can lead to down-regulation of IDO1 and decreased kynurenine levels [59]. However, in our study the dosages administered were not that extremely different and nevertheless the group with the higher total dose of COX-2-inhibitor had the more pronounced changes in the immunological profile. Despite the use of a COX-2-inhibitor we found increased IDO-activity in both groups.

Finally, one might discuss the possible impact of the fact, that more patients in the abdominal hysterectomy used an epidural catheter . However, we know that an epidural catheter is not able to block the stress response to major surgery [60]. If the epidural anesthesia would have had an anti-inflammatory effect, the differences between the groups might have even been bigger than observed in our study.

While taking all these factors into consideration, we are aware of the fact, that our groups are not totally similar. However, when there was a difference between patient demographics or in the anesthetic regimen used, they should have led to smaller differences than what were actually observed, which must be interpreted as extra evidence to support our theory.

## Conclusion

We conclude that surgery and associated anesthesia cause a significant decrease in tryptophan levels, while significantly increasing IDO activity. Both types of surgery produce nearly identical neopterin time curve relationships, with no significant change occurring in either group. However, even though neopterin is unaffected by the severity of surgery, IL-6 responded to surgical invasiveness by revealing a significant increase during major abdominal surgery.
